# *In Vivo* Wall Shear Measurements within the Developing Zebrafish Heart

**DOI:** 10.1371/journal.pone.0075722

**Published:** 2013-10-04

**Authors:** R. Aidan Jamison, Chaminda R. Samarage, Robert J. Bryson-Richardson, Andreas Fouras

**Affiliations:** 1 Laboratory for Dynamic Imaging, Monash University, Melbourne, Victoria, Australia; 2 Department of Mechanical and Aerospace Engineering, Monash University, Melbourne, Victoria, Australia; 3 School of Biological Sciences, Monash University, Melbourne, Victoria, Australia; Bascom Palmer Eye Institute, University of Miami School of Medicine, United States of America

## Abstract

Physical forces can influence the embryonic development of many tissues. Within the cardiovascular system shear forces resulting from blood flow are known to be one of the regulatory signals that shape the developing heart. A key challenge in investigating the role of shear forces in cardiac development is the ability to obtain shear force measurements *in vivo*. Utilising the zebrafish model system we have developed a methodology that allows the shear force within the developing embryonic heart to be determined. Accurate wall shear measurement requires two essential pieces of information; high-resolution velocity measurements near the heart wall and the location and orientation of the heart wall itself. We have applied high-speed brightfield imaging to capture time-lapse series of blood flow within the beating heart between 3 and 6 days post-fertilization. Cardiac-phase filtering is applied to these time-lapse images to remove the heart wall and other slow moving structures leaving only the red blood cell movement. Using particle image velocimetry to calculate the velocity of red blood cells in different regions within the heart, and using the signal-to-noise ratio of the cardiac-phase filtered images to determine the boundary of blood flow, and therefore the position of the heart wall, we have been able to generate the necessary information to measure wall shear *in vivo*. We describe the methodology required to measure shear *in vivo* and the application of this technique to the developing zebrafish heart. We identify a reduction in shear at the ventricular-bulbar valve between 3 and 6 days post-fertilization and demonstrate that the shear environment of the ventricle during systole is constantly developing towards a more uniform level.

## Introduction

It is recognized that the development of the embryonic heart is governed by both genetic and environmental factors. Whilst the genetics of heart development has been extensively investigated (for review see [1]), the role of environmental factors, in particular haemodynamic forces, in cardiac development are poorly understood. Work in the chick model system (reviewed in [2]) has identified a wide range of cardiac defects that result from disrupted flow within the embryonic heart. The zebrafish model, with its advantages for *in vivo* imaging, has been increasingly utilized in the study of cardiac blood flow [3], [4], and has been used to successful identify flow regulated steps in cardiac development such as the growth of the atrioventricular valve in response to retrograde blood flow [5], the trabeculation of the ventricle [6], [7], and the development of the cardiac conduction system [6].

A limiting factor in the detailed examination of the role of shear forces in cardiac development has been the difficulty in determining shear force *in vivo.* A well-established method for experimentally determining the shear force a fluid imparts on a wall is to calculate the gradient of the velocity flow field at the wall. The gradient is commonly determined by measuring the velocity at a single point and assuming a parabolic flow pattern. However, this is an extreme simplification of *in-vivo* flow patterns and omits consideration of the complex cardiovascular geometry and the pulsatile nature of blood flow, both of which are known to result in non-parabolic flow profiles [8]. In the early developing heart a linear flow profile (as used by Hove *et al*. 2003 [4]) may be a more accurate assumption, however, local measurements of velocity at the wall would eliminate assumptions on the flow profile, and improve the accuracy of shear force measurements.

We, and others, have previously described methods utilising particle image velocimetry (PIV) [9] for the accurate measurement of blood flow velocity within the zebrafish heart [4], [10], [11]. PIV has also been utilised for investigations into haemodynamics in the embryonic chicken [12]–[15]. PIV requires a pair of images to be acquired at a specified time interval. Images are divided into sub regions (interrogation windows) and cross correlation used to determine the modal displacement of each sub region between the two frames; this combined with the known time interval gives the instantaneous velocity in each sub region. Critically, in contrast to methods determining velocity from the ejection fraction or a single point velocity measurement, PIV allows measurement of velocity at high spatial resolution within the heart, resulting in a detailed velocity profile, which can be used to determine shear, rather than relying on an assumed linear or parabolic flow profile.

Hove *et al.* [4] and Lu *et al.* [10] used PIV to investigate blood flow velocities within the zebrafish heart. Hove *et al.* [4] investigated the 4.5 days postfertilisation (dpf) zebrafish heart and identified the presence of the heart wall and superficial tissue as interfering with accurate velocity calculation. Lu *et al.* [10] removed the impact of the wall by utilizing defocusing particle tracking velocimetry (PTV) on fluorescent tracer particles, however, the low spatial resolution measurements obtained with PTV are not suitable for wall shear calculation. High-resolution velocity measurements are essential for high-accuracy gradient measures [16] in order to accurately measure shear at the wall. Recently we described the development of cardiac-phase filtering to overcome the detrimental effects the heart wall inflicts on brightfield images used for PIV [11]. This technique retains both high spatial and temporal resolution, however, without the ability to identify the inside wall of the heart, a requirement for determination of wall shear.

Here we describe a method for identifying the location of the heart wall utilising the local signal to noise ratio. The method is performed on high-resolution brightfield image series following cardiac phase filtering. Combining the heart wall information with the data obtained from PIV analysis enables accurate measurement of shear force. This technique provides regional wall shear measurements and meets a critical and unmet need in the investigation of haemodynamic forces within the cardiovascular system. We describe the application of this technique to the developing zebrafish and provide a detailed analysis of the dynamic nature of shear force within the developing heart.

## Methods

The capture of imaging data utilised in this study has been previously described in Jamison *et al.* [11] and is summarised below. The bright field image sequences used in this study are the same as those used in Jamison *et al.* [11].

### Ethics Statement

The treatment and handling of zebrafish was carried out following operating procedures approved by the Monash Animal Services Animal Ethics Committee and adult fish were maintained as part of an approved breeding colony (Licence MAS/2009/02BC). All imaging was carried out under anaesthetic (0.016% 3-amino benzoic acid ethyl ester) to minimise discomfort.

### Imaging

Image acquisition was conducted using an inverted microscope (Leica DM ILM) with a 20× objective lens (Nikon bright field CFI Plan Apo 20×/0.75) coupled via an optical spacer (Leica 2.5×) to a high-speed camera (IDT Y4) resulting in an effective pixel size of 0.28 µm. High-speed brightfield image sequences of the embryonic zebrafish heart were acquired for zebrafish ranging in age from 3 days post fertilization (dpf) to 6 dpf (N = 4 at each stage). Image acquisition lasted for 2 s (approximately 4 cardiac cycles) and was performed at 2000 frames per second, corresponding to ∼800 instantaneous velocity measurement time points per cardiac cycle (depending on fish age). Zebrafish were mounted for viewing of the heart from below and were aligned to minimise flow in the out of plane direction through the ventricular-bulbar valve. Sample positioning was controlled via a 3-axis robotic sample stage (Aerotech; xy resolution 1 µm; z resolution 0.1 µm) and the temperature during image acquisition was 22.5°C±0.5°C.

### PIV

Velocity measurements in this study were performed using an in-house code [17] that has been thoroughly validated [18] and shown to measure motion accurately to 0.03px [17], [19]. Red blood cells were utilised to act as the tracer particles, as has been performed by previous studies [4], [11], [12], [20]. Velocity measurements were calculated using an interrogation window size of 18 µm×18 µm and spacing between measurements of 4.5 µm. The temporal over sampling due to the high-speed acquisition of data was used to improve the velocity measurements by temporally binning measurements into 100 segments of the cardiac cycle and averaging them.

A recent study [21] has considered whether tracer particles are necessary for accurate blood flow measurements. The study found that the accuracy of velocity measurements using red blood cells and tracer particles depends on both the vessel diameter and the magnification. Specifically, for a 100 µm diameter vessel the study indicated that at ‘medium magnification’ (12.5×) both blood cells and tracer particles estimate ∼66% of the actual centerline velocity, and that at ‘high magnification’ (25×) blood cells and tracer particles estimate ∼73% and 79% of the actual centerline velocity, respectively. The study did not consider ‘very high’ magnification, the category the current study would fall into (50×), however it did indicate that as the magnification was increased the velocity underestimation of red blood cells and of tracer particles is reduced.

### Heart Wall Identification

**Figure 1:**
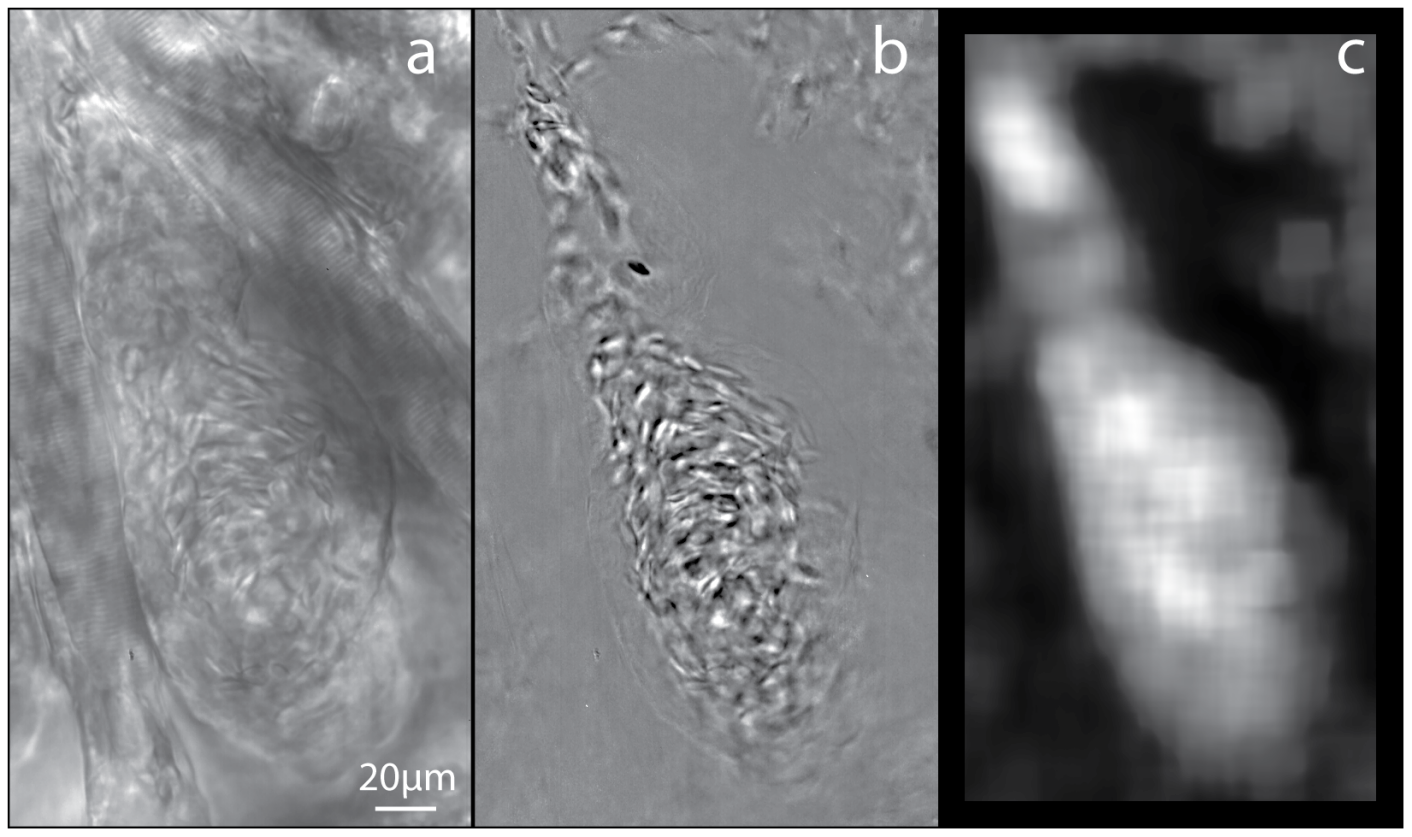
Brightfield images of a 4dpf zebrafish ventricle (a) before and (b) after cardiac-phase filtering. (c) Map of the local signal-to-noise ratio (SNR) after processing (b).

**Figure 2:**
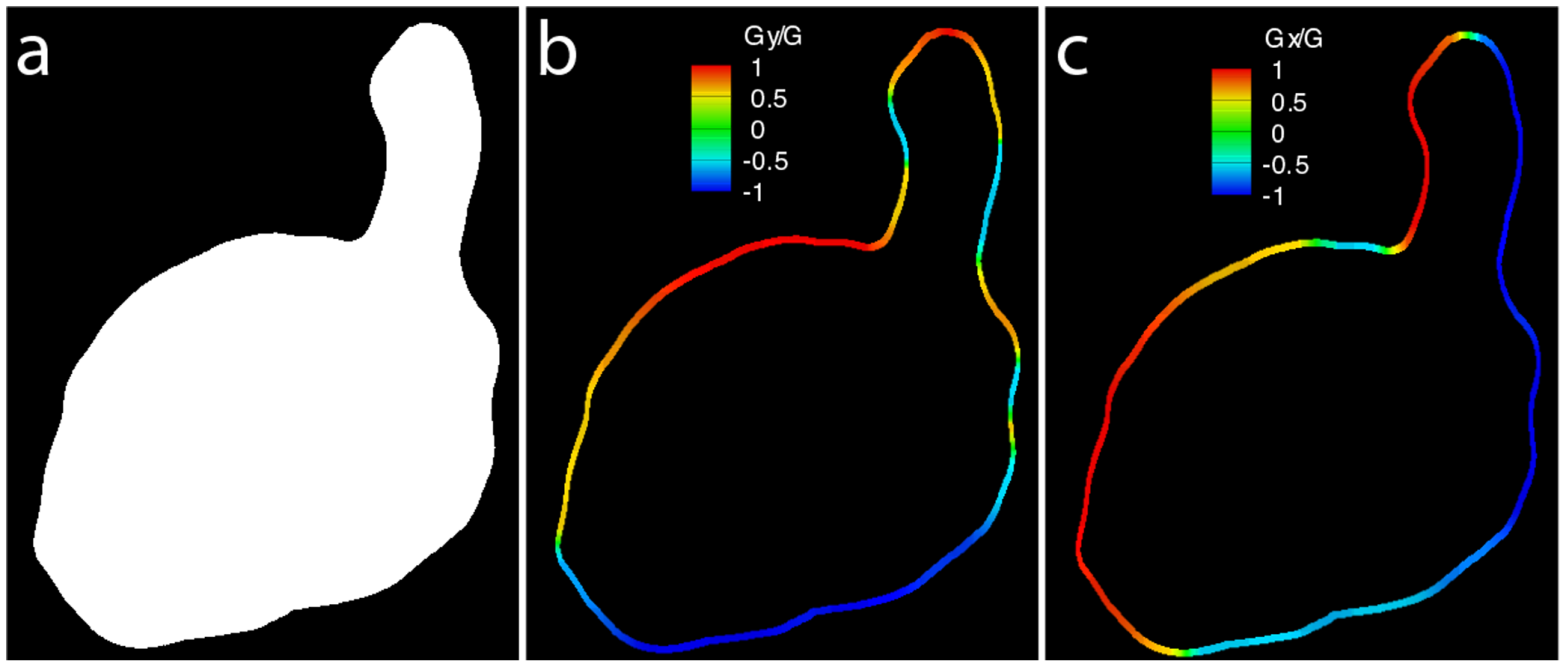
The local signal-to-noise ratio can be used to define the boundary, allowing calculation of the slope of the wall. (a) Binary mask created by thresholding the SNR map. Colourmap of the slope at the wall in (b) the horizontal direction and (c) the vertical direction after the heart wall identification process.

The capture and cardiac-phase filtering of high speed brightfield time-lapse series of zebrafish hearts at 3,4,5, and 6 days post-fertilisation (dpf) has been previously described (Figure 1 a,b; [11]). The automated identification of the inner surface of the heart wall in the unfiltered images (Figure 1a) was not possible due to the inclusion of surrounding structures and insufficient contrast. The cardiac-phase filtering removes static and slow moving structures from the image series, resulting in only the blood flow being evident (Figure 1b). The removal of these structures improves the accuracy of the PIV analysis preventing a decrease in the measured velocity due to the inclusion of static structures in the cross correlation. This also allows for the accurate determination of the inner surface of the heart, which can be defined by the limits of the blood cells. The blood cells were identified utilising the local signal-to-noise ratio (SNR), which was performed on interrogation windows within the image, and is defined as.
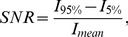
(1)where *I_mean_* is the mean intensity of the interrogation region, and *I_95%_* and *I_5%_* are the values for which only 5% of pixels have a greater or lower intensity, respectively, within the interrogation region. The interrogation region consists of a larger region of pixels surrounding the pixel being evaluated. Several versions of the SNR were evaluated with this version of SNR yielding the best results for the purpose of heart wall detection. This process is performed on every pixel location of the image using a 64×64 pixel interrogation window, resulting in a map of SNR (Figure 1c). A thresholding process is applied to the SNR map to create a binary image, wherein the area that contains red blood cells is white and the rest of the image is black. This binary image defines the area within which to perform PIV and is generally referred to as a mask (Figure 2a). This image can be utilized to determine the location and orientation of the heart wall by means of edge detection routine such as the Sobel operator. The Sobel operator works by applying convolution kernels to the binary image to emphasize regions of high spatial frequency, i.e. in this case, edges of the heart wall.

Edge detection and calculation of the horizontal (**G_x_**) and vertical (**G_y_**) derivatives of the mask (Figure 2) were determined using a Sobel operator with the convolution kernels.
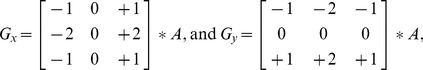
(2)where **A** is the image of the binary mask. These gradients can be combined together to determine the absolute magnitude of the gradient at each point on the wall and the orientation of that gradient. The gradient magnitude, **G**, is determined by



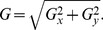
(3)While this process provides the location of the wall, the angle of orientation of the wall (α) is determined using the gradients estimated by the Sobel operator from.
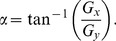
(4)


Using both equations 2 and 3, the slopes in the horizontal direction and the vertical direction at each point on the heart wall are respectively given by.
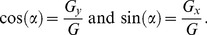
(5)


### Wall Shear Calculation

**Figure 3:**
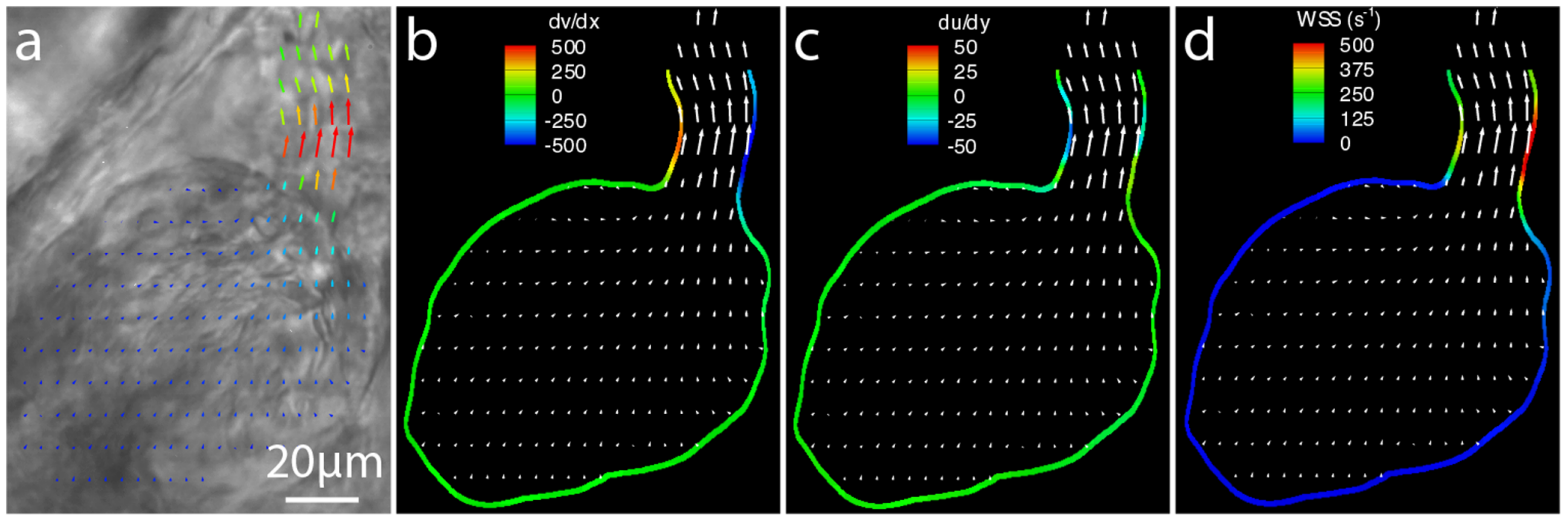
Calculation of the wall shear rate (s ^−1^) from the velocity and the slope of the wall. (a) Brightfield images of a 3dpf zebrafish ventricle during systole with overlaid with velocity vectors calculated using PIV. Contours of (b) dv/dx, (c) du/dy and (d) the calculated wall shear rate (s^−1^) also overlaid with velocity vectors. Shear is concentrated in the region of the ventricular bulbar valve with the majority of the remainder of the heart experiencing comparatively low shear.

The wall shear is calculated using the gradient of the velocity perpendicular to the surface (shear rate) and the viscosity of the fluid. The 2-dimensional wall shear field, 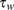, is defined as.
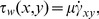
(6)where *μ* is the dynamic viscosity and 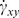 is the 2-dimensional wall shear rate. The wall shear rate is defined as
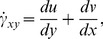
(7)where u and v are the horizontal and vertical components of the velocity vector, respectively. The wall shear rate (s−1) can be calculated using the image gradients in the mask and the corresponding velocity gradient measurements from the PIV measurements (dv/dx and du/dy) by utilizing equation 4 to rewrite equation 6 as



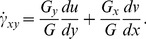
(8)The velocity gradients (*dv/dx* and *du/dy)* are calculated for each measured velocity field (Figure 3b,c) using a second order polynomial fit to the velocity measurements. In order to calculate shear for every position on the wall, the closest 25 velocity gradient measurements were combined using a Gaussian weighting average based on the distance of each measurement from the position of interest on the wall. The averaged velocity gradients were then combined with the slope of the heart wall at the point of interest to calculate the local shear rate (Figure 3d). The shear rate, combined with equation 5 and the dynamic viscosity, can be used to calculate the wall shear stress. Alternatively, the shear rate provides a relative measure of shear force on the heart wall, and removes the need to assume a value for the viscosity of zebrafish blood at each developmental stage. Furthermore, the non-Newtonian characteristics of blood make defining the dynamic viscosity difficult, as it does not have a constant value [22], even in fully developed animals.

## Results and Discussion

### Changes in Wall Shear During Cardiac Development

**Figure 4:**
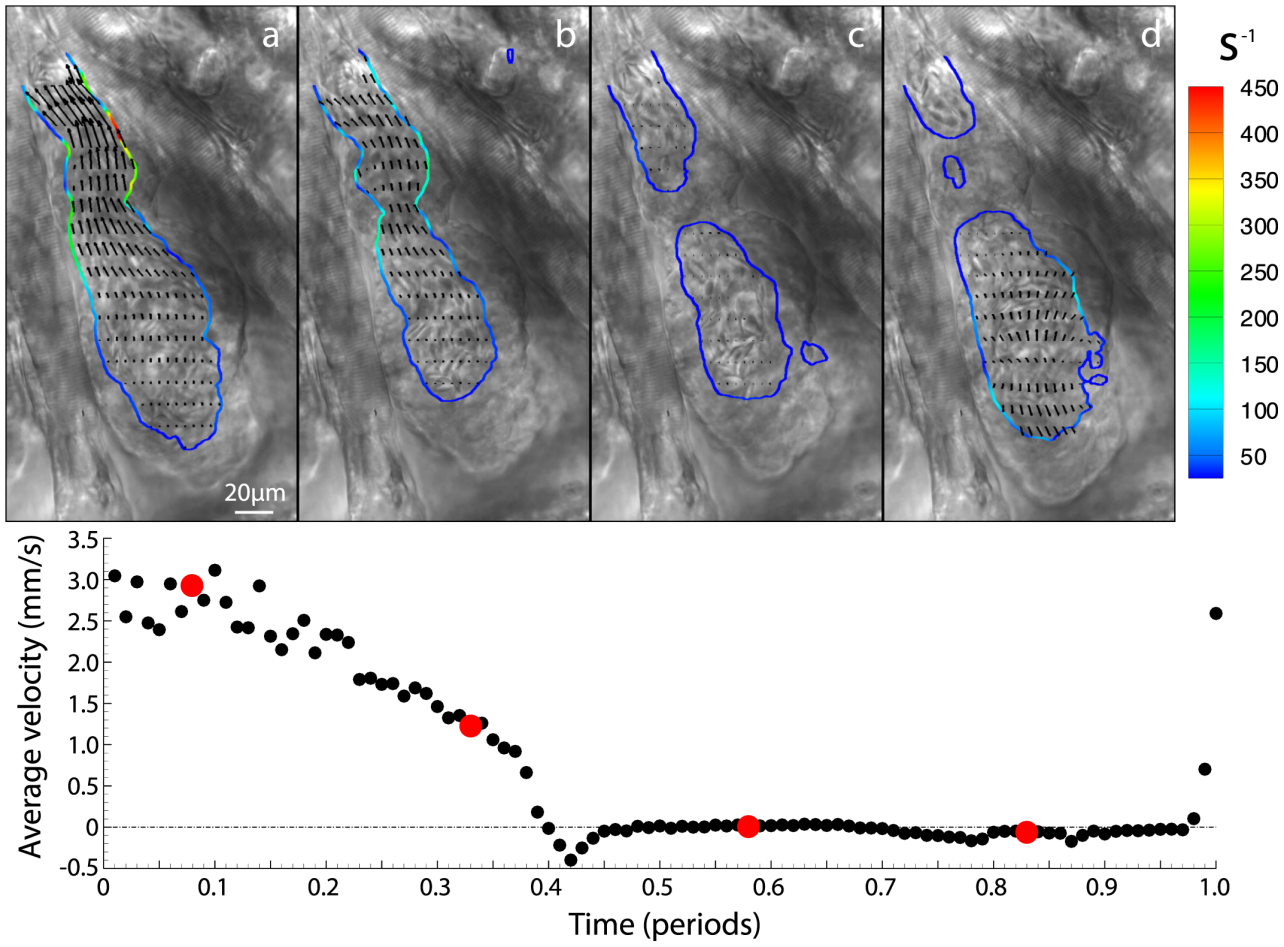
Brightfield images of four equally spaced time points within the cardiac cycle with contours of wall shear (s ^−1^) and vectors of velocity overlaid (Movie S1 provides all 100 times points in the cardiac cycle). For clarity only every second vector is shown in the horizontal direction and every fifth in the vertical direction. Average velocity through the ventricular-bulbar valve is shown below the brightfield images, with red circles indicating the time points corresponding to points (a) – (d).

We applied the methodology we developed to examine shear rate in time-lapse series of heart activity of zebrafish from 3dpf until 6 dpf after which the formation of pigment prevents further analysis (n = 4 at 3,4,5, and 6dpf; [11]). For each image series the shear rate was calculated for 100 time points covering the cardiac cycle (Figure 4, Movie S1). Figure 4a shows systole, when the ventricular-bulbar valve is fully open and the shear is highest. As the cardiac cycle continues we see a reduction in shear and the closing of the valve causing the separation of the red blood cells (inner wall) into two distinct groups (Figure 4b,c). Finally, Figure 4d shows the filling of the ventricle through the atrioventricular valve, with a large portion of the flow being out of plane.

**Figure 5:**
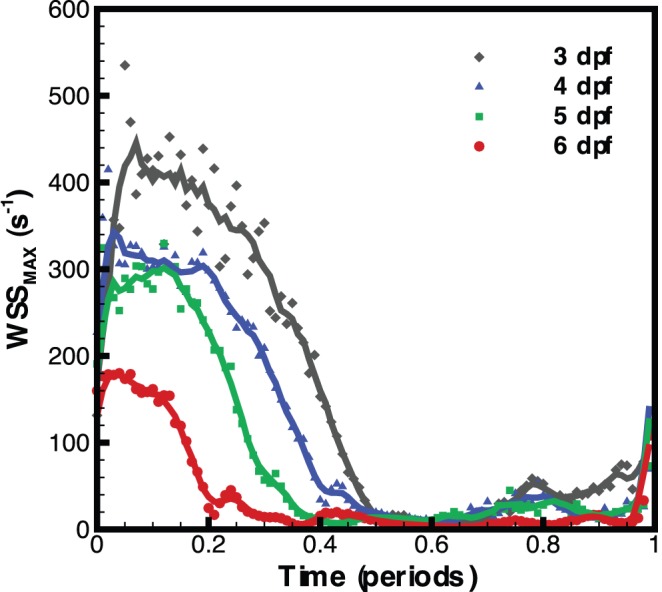
Maximum wall shear rate through the ventricular bulbar valve for each age group, investigated over the entire cardiac cycle. Symbols represent the average for each age group while lines represent a moving average of the data (5 time points).

To examine changes of the wall shear rate at a single point throughout the cardiac cycle we selected the ventricular bulbar (VB) region. The centre of the VB valve was manually identified from the raw images and defined as an analysis zone to allow quantitative analysis. Figure 5 shows the maximum wall shear rate calculated at the VB valve over one full period of the cardiac cycle for each time point investigated; wall shear rate measurements are averaged for each age group. It can be seen that as the fish develops the aperture of the ventricular-bulbar junction increases and the velocity of blood flow decreases [11]. Due to the expansion of the valve and the reduction of velocity, shear is reduced from ∼440 s^−1^ at 3dpf to ∼180 s^−1^ at 6dpf. Additionally, the duration of high wall shear that the valve is exposed to is reduced as the heart develops (from ∼50% to ∼25% of the cardiac cycle).

**Figure 6:**
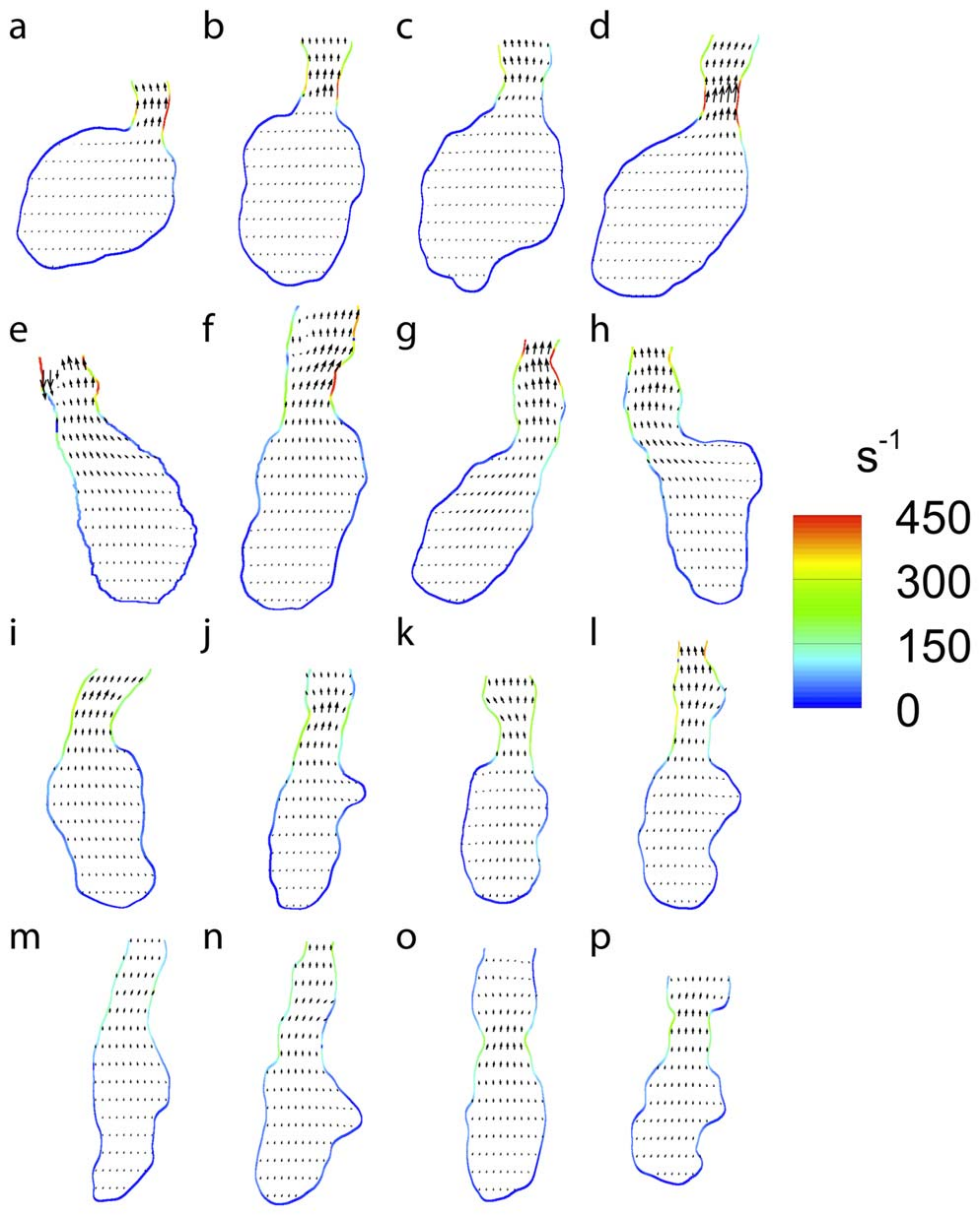
Wall shear rate during peak systole for (a)–(d) 3dpf, (e)–(h) 4dpf, (i)–(l) 5dpf and (m)–(p) 6dpf embryonic zebrafish used in this study. Vectors indicate the magnitude and direction of velocity while colourmap provides the wall shear rate. For clarity only every second vector is shown in the horizontal direction and every fifth in the vertical direction.

The time point in the cardiac cycle that denotes the maximum wall shear rate (peak systole) can be identified from Figure 5. This time point is used to compare the wall shear rate over the ventricle at a similar point in the cardiac cycle for each sample (Figure 6). While the method described here enables velocity and wall shear data to be collected automatically, it cannot identify the boundary of the heart wall as well as a skilled researcher. Therefore, the convenience of the automated system must be weighed against the slightly increased accuracy of a skilled researcher to identify the wall. In order to ensure the highest accuracy for the following peak systole measurements, and to investigate the difference in shear rate measurements for automated and hand masking, a single mask file for each heart at systole was generated by hand.

**Figure 7:**
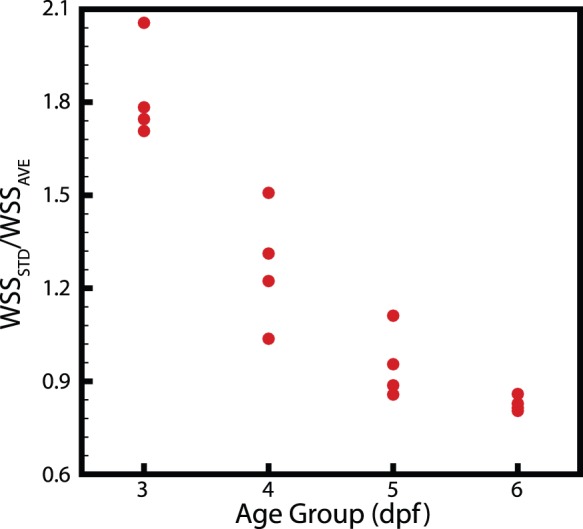
Uniformity measurements of the ventricle during peak systole. A clear trend towards a more uniform shear environment can be seen.

**Figure 8:**
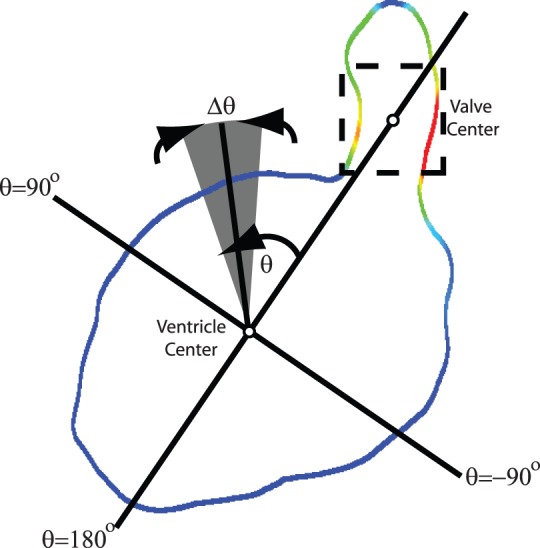
Co-ordinate system used to calculate shear rate at various locations in the ventricle. The ventricular bulbar (VB) valve acts as the reference axis (0° rotation).

Figure 6 shows that the shape of the heart changes substantially as it develops, with the shear rate appearing to become more uniformly distributed over the ventricle wall. In order to test if shear was more evenly distributed as the heart develops we calculated the standard deviation of the shear and divided this by the average of the shear over the entire ventricle (Figure 7). Figure 7 clearly indicates the trend towards a more uniform shear environment as the zebrafish ventricle develops, reinforcing our visual interpretations. In order to examine the distribution of shear along the heart wall we developed a classification system to allow the identification of equivalent regions in different samples at different time points. Two major points are identified, the centre of the VB valve and the centre of the ventricle. The ventricle centre acts as the point of rotation and the valve centre identifies the point of zero rotation (Figure 8).

**Figure 9:**
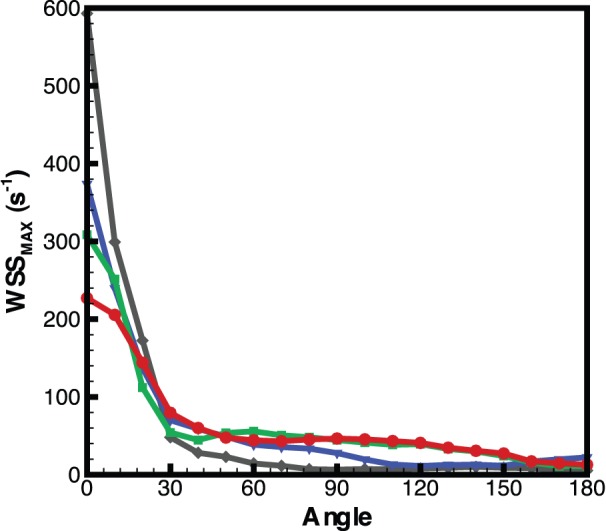
Maximum shear rate measurements within the ventricle corresponding to the co-ordinate system described in Figure 8. Highest shear occurs at 0° (VB valve) and as the fish matures a more uniform shear environment is developed. Measurements are the average for each age group.

Using this classification method the maximum wall shear rate during systole was calculated for each angle of rotation for each age group (Figure 9). Measurements for each fish were averaged for both the positive and negative directions of the angle (treating the heart as being symmetrical), and the average of each age group determined. Figure 9 clearly shows that as the fish matures from 3 to 6dpf that the overall shear it experiences reduces rapidly. The shear rate at the valve is seen to drop substantially as the heart develops, with the shear rate at 10° showing a similar trend. Interestingly, Figure 9 shows that the shear rate at positions away from the valve gradually increase as the heart develops. This demonstrates that the more uniform shear environment that develops in the heart is due to not only the reduction in shear at the valve, but also due to the increases in shear rate at other locations. The increase in shear rate at these locations may be due to changes in heart morphology, in response to high wall shear regions created by flow from the atrium to the ventricle.

A comparison between Figures 5 and 9 enables the difference between the automated masking and the hand masking to be investigated. Measurements through the VB valve provide a good test as they are through a narrow section of the heart, meaning that small changes in the mask file will generate large changes in the measured shear rate. In addition, using measurements at peak systole are the most extreme test case as this is when the highest velocities occur. The shear rate measurements from the automated calculations are within ±10% of the hand generated masks at 3,4, and 5dpf, and within 20% at 6dpf. This is encouraging as it provides us with confidence in the ability of the technique to measure the shear rate at all locations of the heart, while being aware that during the most extreme parts of the cycle, in the more complex regions, there is still good agreement of the results, especially when compared to the variation of the shear stress as the heart develops (a ∼60% decrease in shear rate from 6dpf to 3dpf during peak systole through the VB valve).

### Determination of Shear Stress from Shear Rate

The dynamic viscosity of whole blood is generally considered to range between 3 and 5×10^3^ kg m^−1^ s^−1^, with previous investigations using figures within this range [4], [10], [23]. Using the value of 5×10^3^ kg m^−1^ s^−1^, we estimate peak shear stress values of approximately 25 dyn cm^−2^ at 3 dpf and approximately 17.5 dyn cm^−2^ at 4 and 5 dpf. This value is significantly lower than that previously reported for similarly aged zebrafish, 76 dyn cm^−2^ at 4.5dpf [4]. We believe this difference is due, in part, to the cardiac phase filtering method we have utilized to improve the accuracy of velocity measurements, but most significantly due to our experimental determination of the velocity gradient, in contrast to an assumed linear velocity profile.

## Conclusions

This study is the first to develop an accurate automated measure of wall shear within the developing embryonic zebrafish heart *in vivo*. A technique to accurately determine the location and orientation of the heart wall using a local signal-to-noise ratio analysis is described. PIV measurements of the velocity within the ventricle were acquired at high spatial and temporal resolution, enabling accurate calculation of the velocity gradients. The velocity gradients and heart wall data were combined to determine the wall shear, allowing for detailed investigation of the changes in shear within the ventricle of the developing embryonic heart. We find that as the heart develops, the maximum shear within the heart is reduced (from ∼440 s^−1^ to ∼180 s^−1^), along with the time that it is exposed to this maximum shear (from ∼50% to 25% of the cardiac cycle). Additionally, by comparing the standard deviation of the shear to the average shear in the ventricle, we quantitatively demonstrate that the heart becomes a more uniform shear environment as it develops. The ability to determine the position of the heart wall using the signal-to-noise ratio removes the need for fluorescent markers, allowing the widespread application of this technique without the need to generate transgenic fluorescent strains. Furthermore, this novel capability, to measure wall shear *in vivo,* will enable greater investigation into the effects of haemodynamic forces on not only the heart, but also the entire vascular system.

## Supporting Information

Movie S1 A complete cardiac cycle with contours of wall shear (s^−1^) and vectors of velocity overlaid (100 times points in the cardiac cycle). For clarity only every second vector is shown in the horizontal direction and every fifth in the vertical direction.
(MOV)
